# Crystal structure and Hirshfeld surface analysis of bis­[(eth­oxy­methane­thio­yl)sulfanido](*N*,*N*,*N*′,*N*′-tetra­methyl­ethane-1,2-di­amine)­mercury(II)

**DOI:** 10.1107/S2056989021010549

**Published:** 2021-10-19

**Authors:** Adnan M. Qadir, Sevgi Kansiz, Necmi Dege, Eiad Saif

**Affiliations:** a Department of Chemistry, College of Science, Salahaddin University, Erbil, 44001, Iraq; b Samsun University, Faculty of Engineering, Department of Fundamental Sciences, 55420, Samsun, Turkey; c Ondokuz Mayıs University, Faculty of Arts and Sciences, Department of Physics, 55139, Samsun, Turkey; d Department of Computer and Electronic Engineering Technology, Sanaa Community College, Sanaa, Yemen; e Department of Electrical and Electronic Engineering, Faculty of Engineering, Ondokuz Mayıs University, 55139, Samsun, Turkey

**Keywords:** crystal structure, xanthate, mercury(II), Hirshfeld surface analysis

## Abstract

The title four-coordinate mononuclear complex, [Hg(C_3_H_5_OS_2_)_2_(C_6_H_16_N_2_)] or [Hg(C_3_H_5_OS_2_)_2_(tmeda)] (tmeda: *N*,*N*,*N*′,*N*′-tetra­methyl­ethane-1,2-di­amine), has a distorted tetra­hedral geometry. The Hg^II^ ion is coordinated to two N atoms of the *N*,*N*,*N*′,*N*′-tetra­methyl­ethylenedi­amine ligand and two S atoms from two ethylxanthate xanthate ligands. In the crystal, mol­ecules are linked by weak C—H⋯S hydrogen bonds, forming a two-dimensional supra­molecular architecture in the *ab* plane. The most important contributions for the crystal packing are from H⋯H (59.3%), S⋯H (27.4%) and O⋯H (7.5%) inter­actions.

## Abstract 2

In the crystal, mol­ecules are linked by weak C—H⋯S hydrogen bonds, forming a two-dimensional supra­molecular architecture.

## Chemical context

Xanthates (di­thio­carbonates) attract the inter­est of many researchers in the field of coordination chemistry owing to their anti­dotal, anti­oxidant and anti­tumor activities (Shahzadi *et al.*, 2009 ▸; Perluigi *et al.*, 2006 ▸; Larsson & Oberg, 2011 ▸). These ligands exhibit different coordination modes such as monodentate, isobidentate or anisobidentate. Cellulose xanthate has been used for the separation of alcohols by the chromatographic method (Friebolin *et al.*, 2004 ▸). It has been reported that metal xanthates exhibit cytotoxic activity on human cancer cells and have the ability to inhibit both DNA and RNA viruses *in vitro* (Efrima & Pradhan, 2003 ▸). Mercury represents one of the most toxic heavy metals found in solid and liquid waste from oil refineries and the mining industry. We report herein the synthesis and crystal structure of a new Hg^II^ xanthate containing *N,N,N′,N′*-tetra­methyl­ethylenedi­amine, including the results of a Hirshfeld surface analysis. 
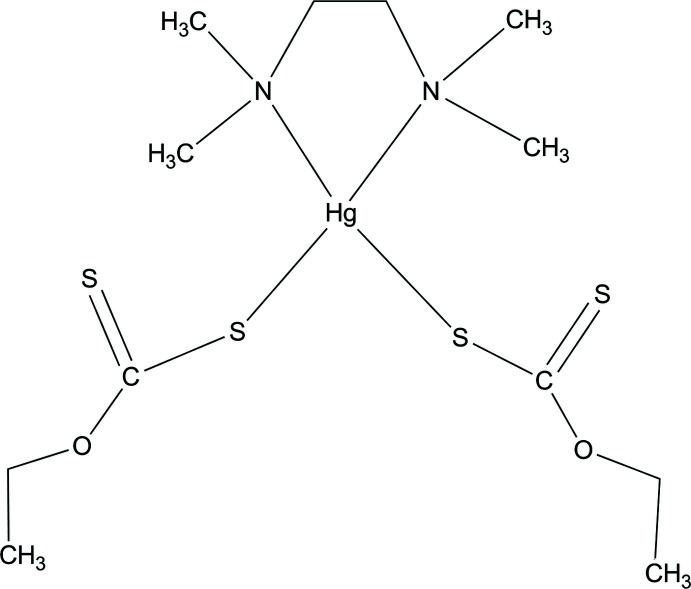


## Structural commentary

**Figure 1:**
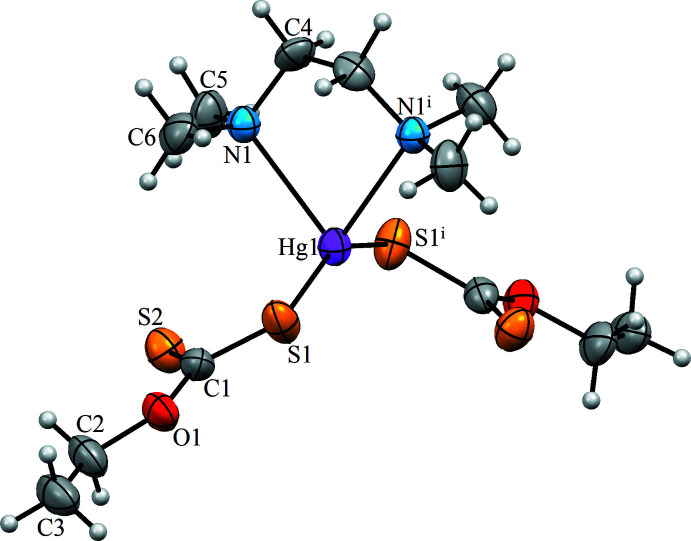
The mol­ecular structure of [Hg(C_3_H_5_S_2_O_1_)_2_(tmeda)], with the atom labelling. Displacement ellipsoids are drawn at the 30% probability level. Symmetry code: (i) −*x* + 1, *y*, −*z* + 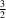 .

The asymmetric unit of the title complex (Fig. 1 ▸) comprises one Hg^II^ ion, one half *N*,*N*,*N*′,*N*′-tetra­methyl­ethylenedi­amine ligand and one ethylxanthate ligand. The Hg^II^ ion is coordinated by two N atoms of the *N,N,N′,N′*-tetra­methyl­ethylenedi­amine ligand and two S atoms from two ethylxanthate xanthate ligands in a distorted tetra­hedral environment. The Hg—N and Hg—S bond lengths (Table 1 ▸) are 2.531 (8) and 2.416 (3) Å, respectively, whereas the bond angles around the central Hg^II^ ion are in the range 73.8 (3)–149.91 (18)°. The bond lengths and angles of the HgN_2_S_2_ coordination units correspond to those in the structures of mixed-ligand Hg^II^ coordination compounds (see *Database survey*). The C1—O1 and C2—O1 bond lengths are 1.355 (11) to 1.460 (12) Å, respectively, although all of the C—O bonds show single-bond character. In the {S_2_C} section of the xanthate ligands, the C1—S1 distance is 1.727 (9) Å, which is typical of a single bond, whereas the C1=S2 distance of 1.633 (10) Å is typical of a carbon-to-sulfur double bond. The C—N and C—C bond lengths in the *N*,*N*,*N*′,*N*′-tetra­methyl­ethylenedi­amine ligand are normal (Qadir *et al.*, 2020 ▸).

## Supra­molecular features

**Figure 2:**
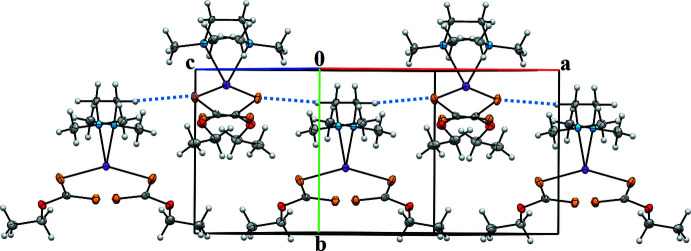
Two-dimensional wave-like structure extending in the *ab* plane formed by hydrogen-bonding inter­actions in [Hg(C_3_H_5_S_2_O_1_)_2_(tmeda)].

In the crystal, there is a weak inter­molecular hydrogen bonding (Table 2 ▸) between S atoms and the H atoms of the methyl­ene groups [C4—H4*B*⋯S1 (*x* − 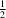 , *y* − 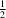 , −*z* + 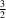 )]. Fig. 2 ▸ illustrates the two-dimensional wave-like structure extending in the *ab* plane formed by hydrogen-bonding inter­actions in [Hg(C_3_H_5_S_2_O_1_)_2_(tmeda)].

## Database survey

A search of the Cambridge Structural Database (CSD, version 5.42, update of November 2020; Groom *et al.*, 2016 ▸) for the title complex revealed five similar complexes: [Hg(C_14_H_26_O_2_S_4_)]_*n*_ (BATXOJ; Cox & Tiekink, 1999 ▸), [Hg(C_5_H_4_NSe)_2_(C_6_H_16_N_2_)] (EKODAK; Sharma *et al.*, 2011 ▸), [Hg(C_6_H_16_N_2_)(C_9_H_13_NS)_2_](PF_6_)_2_ (POTJOY; Tang *et al.*, 2009 ▸), [HgCl(C_7_H_7_S)(C_6_H_16_N_2_)_]_ (TEVQAM; Kräuter *et al.*, 1996 ▸) and [HgCl_2_(C_6_H_16_N_2_)] (ZZZAJM; Htoon & Ladd, 1976 ▸). In BATXOJ, the coordination geometry is distorted tetra­hedral with the independent Hg—S distances being 2.413 (5) and 2.842 (5) Å. The range of S—Hg—S angles is 81.8 (2)–150.8 (3)° with the wider angle involving the more tightly bound S1 atoms. In EKODAK, the corresponding mercury complex adopts a severely distorted tetra­hedral configuration defined by the two monodentate seleno­late and chelating tmeda ligands. The Hg—N bond lengths are in the range 2.573 (17)–2.601 (18) Å. In POTJOY, inter­molecular C—H⋯S hydrogen bonds are important in the crystal packing. Similarly, the mol­ecules are connected to each other *via* C—H⋯S hydrogen bonds in the title complex. In TEVQAM, the Hg—N and Hg—S bond lengths are 2.54 and 2.34 Å, respectively, comparable to those in the title compound.

## Hirshfeld surface analysis

**Figure 3:**
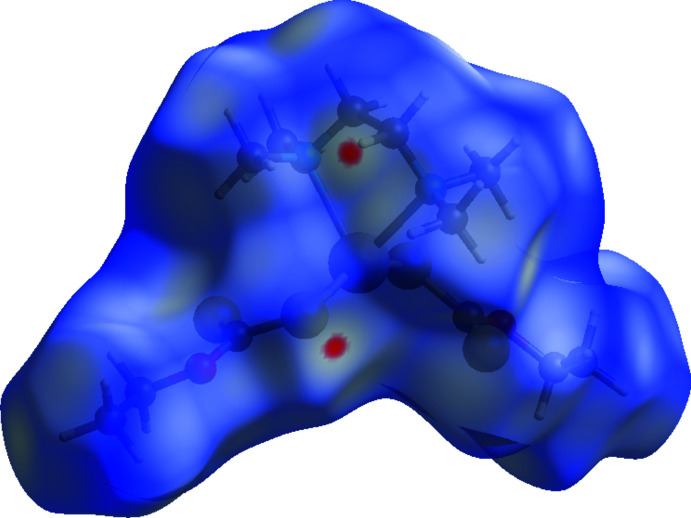
Hirshfeld surface mapped with *d*_norm_.

**Figure 4:**
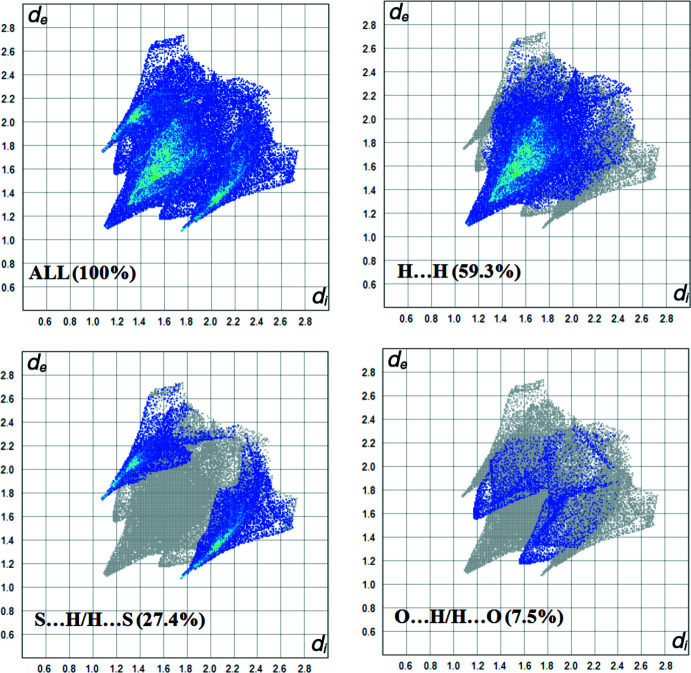
Two-dimensional fingerprint plots for [Hg(C_3_H_5_S_2_O_1_)_2_(tmeda)].

A Hirshfeld surface analysis (Spackman & Jayatilaka, 2009 ▸) was carried out using *CrystalExplorer17.5* (Turner *et al.*, 2017 ▸) to qu­antify the various inter­molecular inter­actions. The Hirshfeld surface mapped over *d*_norm_ is illustrated in Fig. 3 ▸ and the associated two-dimensional fingerprint plots in Fig. 4 ▸. The major contributions to the crystal structure are from H⋯H (59.3%), S⋯H (27.4%) and O⋯H inter­actions (7.5%. The large number of H⋯H inter­actions suggest that van der Waals inter­actions and hydrogen bonding play the major roles in the crystal packing. C⋯H (3.4%) and S⋯O (1.9%) contacts are also observed.

## Synthesis and crystallization

Potassium ethylxanthate (4 mmol, 0.64 g) in hot ethanol (10 mL) was added to a hot solution of Hg(CH_3_CO_2_)_2_ (2 mmol, 0.64 g) in ethanol (10 mL) under stirring. The formed precipitate was filtered off, washed with water and air-dried. The precipitate was suspended in hot ethanol (10 mL) and tetra­methyl­ethylenedi­amine (2 mmol, 0.23 g) was added under stirring. The colour changed to dark brown. The precipitate was filtered off and dried and then recrystallized from ethanol. Brown rods were formed.

## Refinement

Crystal data, data collection and structure refinement details are summarized in Table 3 ▸. C-bound H atoms were positioned geometrically (C—H = 0.96 and 0.97 Å) and refined using a riding model, with *U*_iso_(H) = 1.5*U*_eq_(C) for methyl H atoms and 1.2*U*_eq_(C) for all others

## Supplementary Material

Crystal structure: contains datablock(s) I. DOI: 10.1107/S2056989021010549/ey2008sup1.cif

Structure factors: contains datablock(s) I. DOI: 10.1107/S2056989021010549/ey2008Isup2.hkl

CCDC reference: 2115100

Additional supporting information:  crystallographic information; 3D view; checkCIF report

## Figures and Tables

**Table 1:** Selected bond lengths (Å)

Hg1—S1	2.416 (3)	O1—C1	1.355 (11)
Hg1—N1	2.531 (8)	O1—C2	1.460 (12)
S1—C1	1.727 (9)	N1—C5	1.452 (13)
S2—C1	1.633 (10)	N1—C4	1.479 (13)

**Table 2:** Hydrogen-bond geometry (Å, °)

*D*—H⋯*A*	*D*—H	H⋯*A*	*D*⋯*A*	*D*—H⋯*A*
C4—H4*B*⋯S1^i^	0.97	2.92	3.845 (11)	160

Symmetry code: (i) x-{\script{1\over 2}}, y-{\script{1\over 2}}, -z+{\script{3\over 2}}.

**Table 3:** Experimental details

Crystal data	
Chemical formula	[Hg(C_3_H_5_OS_2_)_2_(C_6_H_16_N_2_)]
*M*_r_	559.18
Crystal system, space group	Orthorhombic, *P* *b* *c* *n*
Temperature (K)	296
*a*, *b*, *c* (Å)	12.235 (7), 8.017 (5), 21.251 (17)
*V* (Å^3^)	2084 (2)
*Z*	4
Radiation type	Mo *K*α
μ (mm^−1^)	7.79
Crystal size (mm)	0.71 × 0.38 × 0.06

Data collection	
Diffractometer	Bruker D8 Quest with Photon II CPADs detector
Absorption correction	Multi-scan (*SADABS*; Krause *et al.*, 2015 ▸)
*T*_min_, *T*_max_	0.041, 0.627
No. of measured, independent and observed [*I* > 2σ(*I*)] reflections	8974, 1946, 1265
*R*_int_	0.139
(sin θ/λ)_max_ (Å^−1^)	0.610

Refinement	
*R*[*F* ^2^ > 2σ(*F* ^2^)], *wR*(*F* ^2^), *S*	0.053, 0.147, 1.00
No. of reflections	1946
No. of parameters	99
H-atom treatment	H-atom parameters constrained
Δρ_max_, Δρ_min_ (e Å^−3^)	1.01, −2.73

Computer programs: *APEX3* and *SAINT* (Bruker, 2017 ▸), *SHELXT2018/2* (Sheldrick, 2015*a* ▸), *SHELXL2017/1* (Sheldrick, 2015*b* ▸), *PLATON* (Spek, 2020 ▸) and *WinGX* (Farrugia, 2012 ▸).

## supplementary crystallographic information

### Crystal data

**Table:**

[Hg(C_3_H_5_OS_2_)_2_(C_6_H_16_N_2_)]	*D*_x_ = 1.782 Mg m^−^^3^
*M**_r_* = 559.18	Mo *K*α radiation, λ = 0.71073 Å
Orthorhombic, *P**b**c**n*	Cell parameters from 379 reflections
*a* = 12.235 (7) Å	θ = 2.0–24.7°
*b* = 8.017 (5) Å	µ = 7.79 mm^−^^1^
*c* = 21.251 (17) Å	*T* = 296 K
*V* = 2084 (2) Å^3^	Rod, brown
*Z* = 4	0.71 × 0.38 × 0.06 mm
*F*(000) = 1088	

### Data collection

**Table:**

Bruker D8 Quest with Photon II CPADs detector diffractometer	1946 independent reflections
Radiation source: Incoatec microfocus source	1265 reflections with *I* > 2σ(*I*)
Detector resolution: 7.4074 pixels mm^-1^	*R*_int_ = 0.139
phi and ω scans	θ_max_ = 25.7°, θ_min_ = 1.9°
Absorption correction: multi-scan (SADABS; Krause *et al.*, 2015)	*h* = −14→12
*T*_min_ = 0.041, *T*_max_ = 0.627	*k* = −6→9
8974 measured reflections	*l* = −25→25

### Refinement

**Table:**

Refinement on *F*^2^	Primary atom site location: dual
Least-squares matrix: full	Hydrogen site location: inferred from neighbouring sites
*R*[*F*^2^ > 2σ(*F*^2^)] = 0.053	H-atom parameters constrained
*wR*(*F*^2^) = 0.147	*w* = 1/[σ^2^(*F*_o_^2^) + (0.0768*P*)^2^] where *P* = (*F*_o_^2^ + 2*F*_c_^2^)/3
*S* = 1.00	(Δ/σ)_max_ < 0.001
1946 reflections	Δρ_max_ = 1.01 e Å^−^^3^
99 parameters	Δρ_min_ = −2.73 e Å^−^^3^
0 restraints	

### Special details

**Table:**

Geometry. All esds (except the esd in the dihedral angle between two l.s. planes) are estimated using the full covariance matrix. The cell esds are taken into account individually in the estimation of esds in distances, angles and torsion angles; correlations between esds in cell parameters are only used when they are defined by crystal symmetry. An approximate (isotropic) treatment of cell esds is used for estimating esds involving l.s. planes.

### Fractional atomic coordinates and isotropic or equivalent isotropic displacement parameters (Å^2^)

**Table:**

	*x*	*y*	*z*	*U*_iso_*/*U*_eq_	
Hg1	0.500000	0.59649 (6)	0.750000	0.0605 (2)	
S1	0.6620 (2)	0.6747 (4)	0.69202 (12)	0.0807 (9)	
S2	0.4722 (2)	0.7975 (5)	0.61444 (14)	0.0815 (8)	
O1	0.6840 (6)	0.8337 (10)	0.5917 (3)	0.0753 (19)	
N1	0.4366 (6)	0.3441 (10)	0.6885 (3)	0.0580 (18)	
C1	0.6025 (8)	0.7760 (12)	0.6290 (4)	0.063 (2)	
C6	0.5057 (9)	0.3204 (18)	0.6319 (5)	0.087 (4)	
H6A	0.502245	0.418481	0.606101	0.131*	
H6B	0.479686	0.226095	0.608454	0.131*	
H6C	0.579968	0.301191	0.644595	0.131*	
C4	0.4462 (8)	0.1997 (13)	0.7314 (5)	0.069 (2)	
H4A	0.441367	0.097608	0.707118	0.083*	
H4B	0.385492	0.201216	0.760778	0.083*	
C3	0.7614 (16)	0.9572 (16)	0.5016 (5)	0.101 (5)	
H3A	0.747525	1.015326	0.462895	0.152*	
H3B	0.795996	0.852358	0.492616	0.152*	
H3C	0.808505	1.023299	0.527706	0.152*	
C5	0.3232 (9)	0.3668 (17)	0.6701 (5)	0.090 (4)	
H5A	0.280086	0.390950	0.706757	0.136*	
H5B	0.296750	0.266672	0.650617	0.136*	
H5C	0.317899	0.457818	0.640942	0.136*	
C2	0.6563 (11)	0.9271 (15)	0.5349 (5)	0.091 (4)	
H2A	0.621592	1.031988	0.545653	0.109*	
H2B	0.606886	0.863221	0.508546	0.109*	

### Atomic displacement parameters (Å^2^)

**Table:**

	*U*^11^	*U*^22^	*U*^33^	*U*^12^	*U*^13^	*U*^23^
Hg1	0.0589 (4)	0.0689 (4)	0.0536 (3)	0.000	0.0023 (2)	0.000
S1	0.0618 (15)	0.113 (2)	0.0676 (13)	−0.0248 (17)	−0.0088 (11)	0.0231 (15)
S2	0.0674 (17)	0.096 (2)	0.0811 (16)	0.0098 (16)	−0.0023 (13)	0.0141 (16)
O1	0.082 (5)	0.085 (5)	0.059 (3)	−0.011 (4)	0.009 (3)	0.013 (3)
N1	0.056 (5)	0.064 (5)	0.054 (4)	0.000 (4)	0.002 (3)	0.000 (3)
C1	0.075 (6)	0.059 (6)	0.056 (5)	−0.015 (5)	0.004 (4)	0.000 (4)
C6	0.103 (9)	0.088 (9)	0.071 (6)	−0.008 (8)	0.012 (5)	−0.024 (6)
C4	0.057 (6)	0.067 (6)	0.084 (6)	−0.023 (6)	−0.006 (5)	−0.010 (5)
C3	0.142 (13)	0.083 (10)	0.079 (7)	0.005 (8)	0.037 (7)	0.015 (7)
C5	0.075 (8)	0.123 (11)	0.073 (6)	−0.013 (7)	−0.019 (6)	−0.003 (6)
C2	0.115 (10)	0.090 (8)	0.067 (6)	0.005 (8)	0.018 (7)	0.025 (5)

### Geometric parameters (Å, º)

**Table:**

Hg1—S1	2.416 (3)	C6—H6C	0.9600
Hg1—S1^i^	2.416 (3)	C4—C4^i^	1.53 (2)
Hg1—N1	2.531 (8)	C4—H4A	0.9700
Hg1—N1^i^	2.531 (8)	C4—H4B	0.9700
S1—C1	1.727 (9)	C3—C2	1.49 (2)
S2—C1	1.633 (10)	C3—H3A	0.9600
O1—C1	1.355 (11)	C3—H3B	0.9600
O1—C2	1.460 (12)	C3—H3C	0.9600
N1—C5	1.452 (13)	C5—H5A	0.9600
N1—C4	1.479 (13)	C5—H5B	0.9600
N1—C6	1.481 (13)	C5—H5C	0.9600
C6—H6A	0.9600	C2—H2A	0.9700
C6—H6B	0.9600	C2—H2B	0.9700
			
S1—Hg1—S1^i^	149.91 (18)	N1—C4—H4A	109.1
S1—Hg1—N1	101.26 (18)	C4^i^—C4—H4A	109.1
S1^i^—Hg1—N1	102.70 (19)	N1—C4—H4B	109.1
S1—Hg1—N1^i^	102.70 (19)	C4^i^—C4—H4B	109.1
S1^i^—Hg1—N1^i^	101.26 (18)	H4A—C4—H4B	107.8
N1—Hg1—N1^i^	73.8 (3)	C2—C3—H3A	109.5
C1—S1—Hg1	99.9 (3)	C2—C3—H3B	109.5
C1—O1—C2	119.2 (9)	H3A—C3—H3B	109.5
C5—N1—C4	109.8 (9)	C2—C3—H3C	109.5
C5—N1—C6	110.1 (8)	H3A—C3—H3C	109.5
C4—N1—C6	110.8 (9)	H3B—C3—H3C	109.5
C5—N1—Hg1	109.3 (7)	N1—C5—H5A	109.5
C4—N1—Hg1	106.4 (5)	N1—C5—H5B	109.5
C6—N1—Hg1	110.3 (6)	H5A—C5—H5B	109.5
O1—C1—S2	124.9 (7)	N1—C5—H5C	109.5
O1—C1—S1	107.7 (7)	H5A—C5—H5C	109.5
S2—C1—S1	127.4 (5)	H5B—C5—H5C	109.5
N1—C6—H6A	109.5	O1—C2—C3	106.0 (11)
N1—C6—H6B	109.5	O1—C2—H2A	110.5
H6A—C6—H6B	109.5	C3—C2—H2A	110.5
N1—C6—H6C	109.5	O1—C2—H2B	110.5
H6A—C6—H6C	109.5	C3—C2—H2B	110.5
H6B—C6—H6C	109.5	H2A—C2—H2B	108.7
N1—C4—C4^i^	112.7 (7)		
			
C2—O1—C1—S2	1.9 (13)	C5—N1—C4—C4^i^	−162.2 (10)
C2—O1—C1—S1	−179.2 (8)	C6—N1—C4—C4^i^	75.9 (12)
Hg1—S1—C1—O1	178.2 (6)	Hg1—N1—C4—C4^i^	−44.0 (11)
Hg1—S1—C1—S2	−3.0 (7)	C1—O1—C2—C3	−174.4 (9)

Symmetry code: (i) −*x*+1, *y*, −*z*+3/2.

### Hydrogen-bond geometry (Å, º)

**Table:**

*D*—H···*A*	*D*—H	H···*A*	*D*···*A*	*D*—H···*A*
C4—H4*B*···S1^ii^	0.97	2.92	3.845 (11)	160

Symmetry code: (ii) *x*−1/2, *y*−1/2, −*z*+3/2.
